# Graphene Oxide: Graphene Quantum Dot Nanocomposite for Better Memristic Switching Behaviors

**DOI:** 10.3390/nano10081448

**Published:** 2020-07-24

**Authors:** Lei Li

**Affiliations:** 1 HLJ Province Key Laboratories of Senior-Education for Electronic Engineering, Heilongjiang University, Harbin 150080, China; lileidtk@hlju.edu.cn; 2 Research Center for Fiber Optic Sensing Technology National Local Joint Engineering, Heilongjiang University, Harbin 150080, China

**Keywords:** tristable memristic switching, all-inorganic multi-bit memory, charge-trap memristor, GO:GQDs nanocomposite

## Abstract

Tristable memristic switching provides the capability for multi-bit data storage. In this study, all-inorganic multi-bit memory devices were successfully manufactured by the attachment of graphene quantum dots (GQDs) onto graphene oxide (GO) through a solution-processable method. By means of doping GQDs as charge-trapping centers, the device indium-tin oxide (ITO)/GO:0.5 wt%GQDs/Ni revealed controllable memristic switching behaviors that were tunable from binary to ternary, and remarkably enhanced in contrast with ITO/GO/Ni. It was found that the device has an excellent performance in memristic switching parameters, with a SET1, SET2 and RESET voltage of −0.9 V, −1.7 V and 5.15 V, as well as a high ON2/ON1/OFF current ratio (10^3^:10^2^:1), and a long retention time (10^4^ s) together with 100 successive cycles. The conduction mechanism of the binary and ternary GO-based memory cells was discussed in terms of experimental data employing a charge trapping-detrapping mechanism. The reinforcement effect of GQDs on the memristic switching of GO through cycle-to-cycle operation has been extensively investigated, offering great potential application for multi-bit data storage in ultrahigh-density, nonvolatile memory.

## 1. Introduction

The memristor (memory-resistor) is a fourth fundamental passive circuit element, and its resistance can drastically vary with a minor change in input [1,2]. It cannot be modeled with the capacitor (C), resistor (R), or inductor (L), not because they are fundamental, but because memristors are composite resistors that rely on active hysteresis to switch between low and high resistance states. The memristor’s potential for phenomenal computing is in no way diminished by the negative assessment of its qualifications as a fundamental device [3,4]. With remarkable features, such as scalability, low power consumption and dynamic responses, it has recently been anticipated to take a massive leap in revolutionizing the electronic circuits for nonvolatile memory and programmable logic [5,6,7].

Inorganic, polymeric, and organic materials predominantly have been found to exhibit memristor properties [8,9,10]. Most of them, nevertheless, are suitable for binary systems based on electrical bistability. Namely, the data storage capacity is limited in 2^n^. It is essential for developing highly integrated memory devices [11,12,13,14], that multi-bit operation is confronted with currently available commercial memory devices to allow the capacity for more than one bit (a digital state of ‘0’ or ‘1’) in a particular memory cell.

Graphene oxide (GO) is a layered material consisting of hydrophilic, oxygenated graphene sheets. The functional groups presented on the basal planes and edges of GO are reactive sites for physical/chemical bonding with polymers and alter the van der Waals interactions, preventing them from aggregation [15,16,17]. Due to their presence, GO can be stably dispersed into various polar solvents, like water. Apart from its lower cost of fabrication, higher scalability, lightweight and environment-friendly natures, the advantages of GO-based memory include features, such as flexibility, solution processing and 3D-stacking capability [18,19,20,21]. Graphene quantum dots (GQDs) bear unique properties of high electron mobility, excellent solubility and easy functionalization [22,23,24,25,26]. Particularly, carbon nanomaterials have acted as the charge trapping centers to realize the memory effect [27,28]. The memristic parameter indices, such as the ON/OFF current ratio, power consumption, retention stability and reproducibility have been effectively tunable by way of the blending approach. Considering these benefits, it is reasonable to expect GQDs doping to enhance GO as excellent memristic switching materials for multi-bit data storage.

It still needs solutions for multi-bit memory devices to surmount some technical obstacles, such as complex fabrication processes, or multilayer stacked structures [12,29,30,31,32,33]. Herein, all-inorganic memristic switching devices based on GO were fabricated by means of a simple solution-processable approach, which transformed behaviors from binary to ternary memory by doping GQDs. Fourier transform infrared (FTIR) spectroscopy, Raman spectroscopy, thermogravimetric analysis (TGA), as well as scanning electron microscopy (SEM) characterization techniques were performed for the nature assessment of GO and GO:GQDs nanocomposite system. Great efforts have been made to establish the memristic switching characteristics of GO-based nanocomposites, and to interrogate the effect of GQDs introduced into such systems. The study included an evaluation of multi-bit memristic switching behaviors and made a comparison between GO and its nanocomposites, comprising the cycle-to-cycle and device-to-device performance of indium-tin oxide (ITO)/GO/Ni and ITO/GO:GQDs/Ni devices.

## 2. Materials and Methods

### 2.1. Preparation of GO:GQDs Memory Devices

The GO powder and GQDs aqueous dispersion were purchased from Tanfeng Tech. Inc. (Suzhou, China) and utilized without further purification. The thickness, diameter and number of GO layers separately ranged from 3.4 nm to 7 nm, from 10 μm to 50 μm, and from 6 to 10. Moreover, the specific area ranged from 100 m^2^/g to 300 m^2^/g. Pristine GQDs aqueous solution has a concentration of 1 mg/mL. The thickness, diameter and purity of GQDs is 15 nm, 0.5 nm to 2.0 nm, and ~80% (with a small amount of NaOH to regulate the pH value of the solution), respectively. Both GO and GO:GQDs nanocomposite films, fabricated as below, played the role of the memory layer. They were first prepared by dissolving 50 mg of GO powder in 10 mL of distilled water. The GO solution (5 mg/mL) was stirred overnight to guarantee that it dissolved well. Next, an appropriate amount of the pristine GQDs aqueous solution was added into the dissolved GO solution to generate GO:GQDs nanocomposites with content of GQDs 0.5 wt%, 2.5 wt%, and 5.0 wt% before the prepared GO:GQDs nanocomposite solution was stirred for another 5 h to ensure the homogeneous solution. The glass substrate coated with ITO was ultrasonically washed by being rinsed in acetone, methyl alcohol, and absolute ethyl alcohol, respectively. Then GO and its nanocomposites were blended with different content of GQDs in deionized water, which was separately spin-coated on ITO at 6000 rpm for 60 s and then dried at 100 °C for 2 h to allow any excess solvent to evaporate. Finally, the top Ni electrode was deposited on the top of the memory layer to form a metal-insulator-metal (MIM) structure with an active area of 1 × 1 mm^2^. Ni electrodes were deposited onto films (coated on the substrates) by ZZ-450A Vacuum Thermal Evaporator (Beiyi Innovation Vaccum Technology; Beijing, China) at a pressure approaching 10^−5^ Torr.

### 2.2. Characterization

Different characterization techniques were used to confirm the formation of GO:GQDs nanocomposites in conjunction with nanocomposite film characterization. FTIR spectroscopy (Foss DS 2500 Infrared Spectrometer; Hillerød, Denmark), swept from 400 cm^−1^ to 4000 cm^−1^, was recorded. Additionally, Raman spectroscopy (inVia-Reflex; Renishaw, England) was employed to achieve the structure information of GO and GO:GQDs nanocomposites, scanned from 100 cm^−1^ to 3200 cm^−1^, with the 532 nm laser-source and the power of the laser 50 mW. Through thermogravimetric analysis and derivative thermogravimetry (TGA-DTG) (TA Instruments; New Castle, DE, USA) analysis, the thermal stability of GO and its nanocomposites was conducted under N_2_ atmosphere from room temperature growing up to 400 °C at a heating rate of 10 °C/min. Scanning electron microscopy (SEM) (Themoscientific; Waltham, MA, USA) images were obtained at 20 kV and detected under the condition of magnification of 100,000 and working distance (WD) of 10.6 mm, dwell of 5 μs and spot of 10, and PW of 2.7 nm. Transmission electron microscopy (TEM) and high-resolution (HR)-TEM images for GO were characterized by a JEM-2100 TEM (JOEL; Tokyo, Japan) operated at 200 kV. The electrical measurements of ITO/GO/Ni and ITO/GO:GQDs/Ni were fulfilled by a semiconductor parameter analyzer (Keithley 4200; Solon, OH, USA). At room temperature, all electrical experiments were conducted without any device encapsulation in the air.

## 3. Results and Discussions

### 3.1. FTIR Analysis

**Figure 1:**
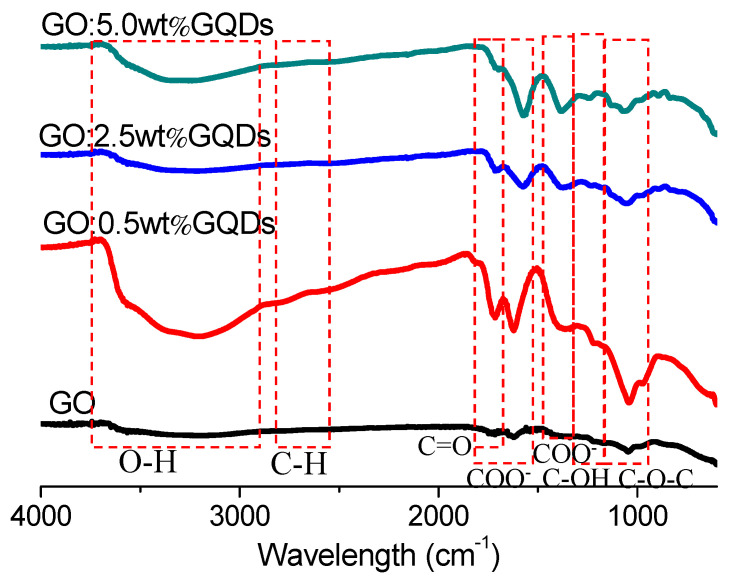
FTIR spectra of GO, and GO:GQDs nanocomposites.

To gain insight into the chemical structures for GO and GO:GQDs nanocomposites, FTIR spectroscopy was used to verify functional group existence (Figure 1) [34]. The peak at 3185 cm^-1^ was indicated by hydroxyl group (O–H), stretching vibrations of GO when prominent peaks for epoxy (C–O–C) and carboxyl groups (–COOH) were observed at 955 cm^−1^ to 1100 cm^−1^, and 1500 cm^−1^ to 1800 cm^−1^. The absorption bands at 1725 cm^−1^, 1163 cm^−1^ and 1338 cm^−1^ were attributed to the stretching of the C=O, deformation of OH and stretching of C–O bonds of COOH groups. The stretching of C–O bond, relevant to the epoxide groups, could be seen at 1046 cm^−1^ when the absorption of C–H stretching could be seen at 2860 cm^−1^. It was found that there was a series of characteristic absorption peaks in the spectra of GO:GQDs nanocomposites. The peaks (Table 1) were associated with the C–O–C, C–OH, C–C, C=O, and OH groups. In the GO:GQDs nanocomposites, a broad and intense hydroxyl (O–H) peak was responsible for the peak at 3208 cm^−1^, 3225 cm^−1^ and 3240 cm^−1^, respectively. The strong C=O peak at around 1710 cm^−1^ was a result of stretching vibrations of carboxylic acid and carbonyl moieties, which decreased with the incremental content of GQDs. These clearly indicated that through COO bonding, GQDs were well attached to the GO surface [35]. The FTIR observation confirmed the production of GO:GQDs nanocomposite formation.

### 3.2. Raman Spectroscopy

**Figure 2:**
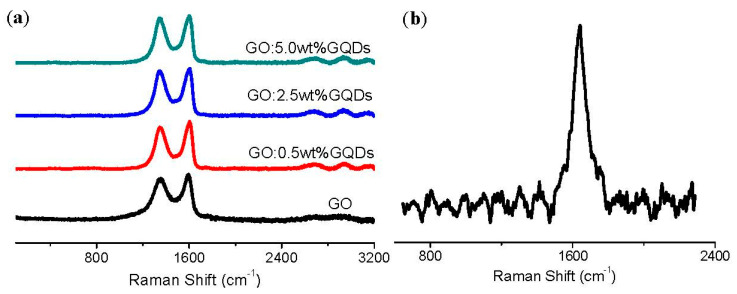
(**a**) Raman spectra of GO and its nanocomposites with different content of GQDs 0.5 wt%, 2.5 wt%, and 5.0 wt%, respectively; (**b**) Raman spectrum of GQDs aqueous dispersion.

Raman spectroscopy was employed to structurally characterize GO and GO:GQDs nanocomposites with 0.5 wt%, 2.5 wt%, and 5.0 wt% GQDs (Figure 2). Two distinct peaks in the spectrum were relative to the broad disorder D-band at 1347 cm^−1^ and an in-plane vibrational G-band at 1591 cm^−1^. The D band related to the mode of the k-point photons of A1g symmetry of hybridized carbon atoms accords with sp^3^ defects, whereas the G band conforms to the E2g phonon of sp^2^ carbon atoms frequencies, connected with the defect density, disorder, edge structure and smoothness [36]. For GO, the intensity ratio of the D band to G band (*I*_D_/*I*_G_) was 0.92, which signified that GO was graphitized at low levels on account of oxygen-containing functional groups [37]. It proved the existence of structural defects and disorder in the sp^2^ network of GO. The estimated intensity ratio of the D band to G band *I*_D_/*I*_G_ for GO:0.5 wt%GQDs nanocomposite was 0.90 while that for GO:5.0wt%GQDs nanocomposite was 0.96, which made clear that GO:5.0 wt%GQDs nanocomposite had more defects and disorders. The broad 2D peak at 2703 cm^−1^ manifested that the GO sample had a small number of layers [38]. Three low-intensity peaks located at approximately 456 cm^−1^, 515 cm^−1^, and 633 cm^−1^, were observed in GO:GQDs nanocomposites and they could be exclusively attributed to the E_g_, F_2g_ and A_1g_ modes of GQDs. Raman spectroscopy gains insight into the ordered and disordered crystal structures of carbon materials. Figure 2b shows the Raman spectrum of the GQDs aqueous dispersion. It indicated a G band at 1643 cm^−1^, which was a common signature of the first-order scattering of E2g phonon from sp^2^ carbon graphitic lattice. The D band was very weak, indicating that the arrangement of the GQD layer was orderly.

### 3.3. Thermal Properties

**Figure 3:**
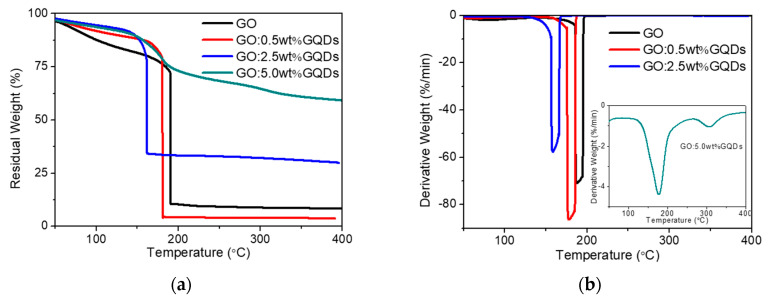
(**a**) Thermogravimetric analysis (TGA) and (**b**) derivative thermogravimetry (DTG) of GO as well as its nanocomposites.

The thermal stability of GO and GO:GQDs nanocomposites were investigated by TGA in N_2_ atmosphere (Figure 3). GO was thermally unstable and started to lose mass upon heating below 100 °C because of the adsorbed water molecules. There were two significant steps in mass around the range from 165 °C to 200 °C, which were assigned to the elimination of oxygenated functional groups of GO. A weak mass loss in the range of 250–400 °C was related to the elimination of more stable functional groups. The residual weight of about 8% was obtained, exhibiting that most of GO was burned into volatile gases. Compared with GO, the GO:GQDs nanocomposites showed lower weight loss at the three stages. This was attributed to the presence of more stable oxide groups on the GO:GQDs nanocomposite surface than GO [35]. The remaining weight at 400 °C was about 4%, 30%, and 59% for GO:0.5 wt%GQDs, GO:2.5 wt%GQDs and GO:5.0 wt%GQDs nanocomposites, respectively. The addition of 0.5 wt% GQDs adversely affected the thermal stability of GO when it might be derived from the interaction between GO and GQDs.

### 3.4. Memory Characteristics of ITO/GO:GQDs/Ni

**Figure 4:**
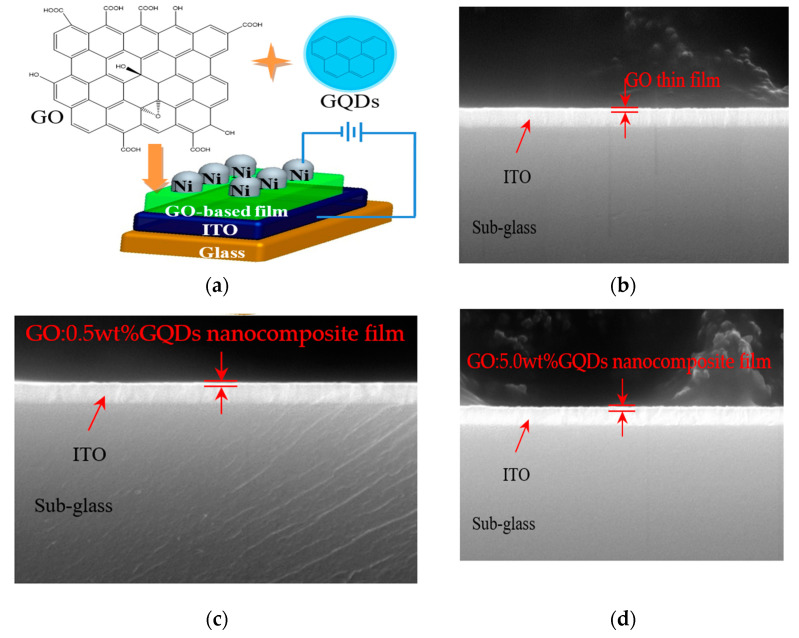
(**a**) Sandwiched scheme of ITO/GO-based/Ni together with structure of chemical component GO and GQDs; (**b**–**d**) SEM images for the cross-sectional characterization of GO, GO:0.5 wt%GQDs, and GO:5.0 wt%GQDs nanocomposite thin films spin-coated onto ITO/glass substrates, respectively.

**Figure 5:**
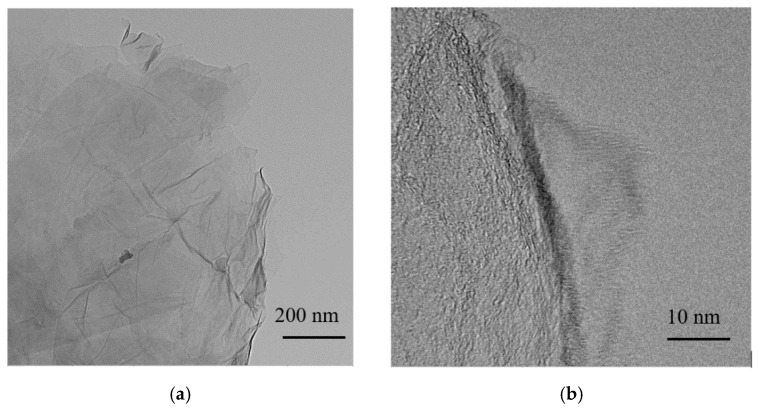
(**a**) Transmission electron microscopy (TEM) and (**b**) high-resolution (HR-TEM images of GO made by magnetically stirring.

A metal/insulator/metal configuration ITO/GO-based film/Ni of memory devices, together with the chemical structures of GO and GQDs can be observed in Figure 4a, which was sandwiched between ITO and Ni electrodes. The cross-sectional profiles of GO and GO:GQDs nanocomposite films were studied by SEM (Figure 4b–d). TEM and HR-TEM images of GO made by magnetically stirring on Cu grids can be observed in Figure 5a,b.

**Figure 6:**
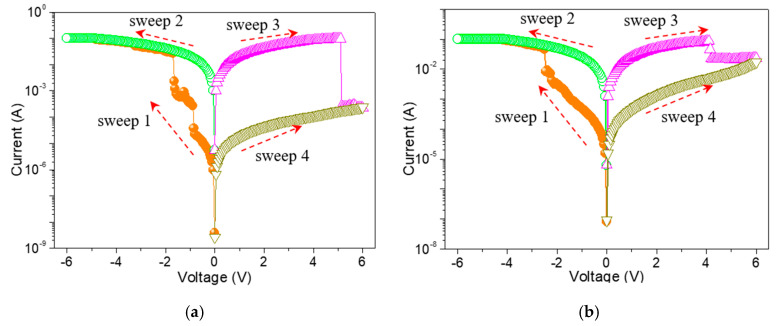
Memristic switching characteristics for (**a**) ITO/GO:0.5 wt%GQDs/Ni and (**b**) ITO/GO/Ni.

**Figure 7:**
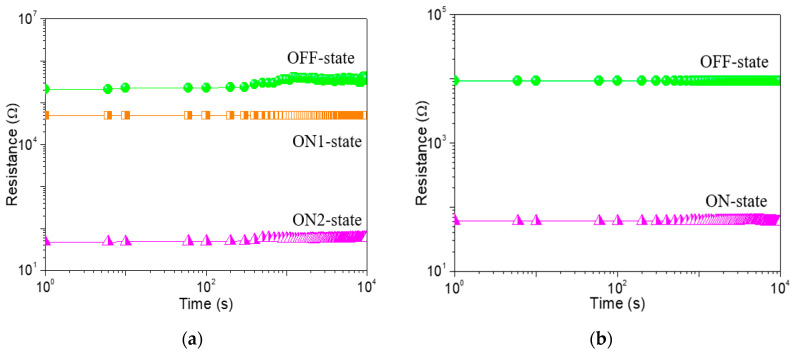
Retention property of (**a**) ITO/GO:0.5 wt%GQDs/Ni device and (**b**) ITO/GO/Ni. The resistance value was read at a constant voltage of −0.1 V.

To study the memristic switching effect of GO-based films, the current-voltage (*I*-*V*) characteristics of ITO/GO-based film/Ni were tested by direct current DC voltage sweep. During the test, the bias was applied to Ni while ITO was grounded. An ITO/GO:GQDs/Ni memory device with GQDs concentration of 0.5 wt% was fabricated to analyze its behaviors. Two obvious high current jumps arose at −0.9 V (*V*_SET1_) and −1.7 V (*V*_SET2_) for the tristable memristic switching behaviors (Figure 6a) when the negative voltage was swept from 0 V to −6 V (sweep 1). Three different resistance levels with a current ratio of about 10^3^:10^2^:1 were obtained, regarded as three storage states: “0” (OFF-state or high resistive state (HRS)), “1” (ON1-state or intermediate resistive state (IRS)) and “2” (ON2-state or low resistive state (LRS)). Once the device was written to be the “2” state, positive sweeping (*V*_RESET_ = 5.14 V) could recover it to the initial “0” state (sweep 3). To demonstrate the nonvolatile nature of the ternary data storage, a small bias voltage (−0.1 V) was used to read the “0”, “1” and “2” states for 10^4^ seconds (Figure 7a) that were kept stable without obvious degradation.

In order to give an insight into the conduction mechanism of the tristable memristic switching based on GO: 0.5 wt%GQDs nanocomposite, memristic switching characteristics of ITO/GO/Ni were implemented under a compliance current (*I*_CC_) of 100 mA (Figure 6b). Initially, it recorded that the current gradually increased on the condition of the incremental voltage from 0 V to −2.2 V. However, an abrupt growth in current at the SET voltage (*V*_SET_) of −2.25 V contributed to the translation of the device from OFF-state to ON-state. The device was stably preserved in ON-state during the subsequent negative voltage sweep. Next, it was switched from ON-state to OFF-state with positive RESET voltage (*V*_RESET_ = 4.15 V). For this binary memory device, OFF-state remained intact against positive sweep up to 6 V. Counting on its bistable memristic switching, the resistance ratio of the device between OFF-state and ON-state was above 10^2^. The OFF-state and ON-state retention of the ITO/GO/Ni memory device without embedded GQDs (Figure 7b) exhibited the device could not be switched between ON-state and OFF-state by applying a constant reading bias of −0.1 V. Taking the bistable memristic switching of GO into consideration, the ternary memory of GO:GQDs nanocomposite was associated with GQDs.

**Figure 8:**
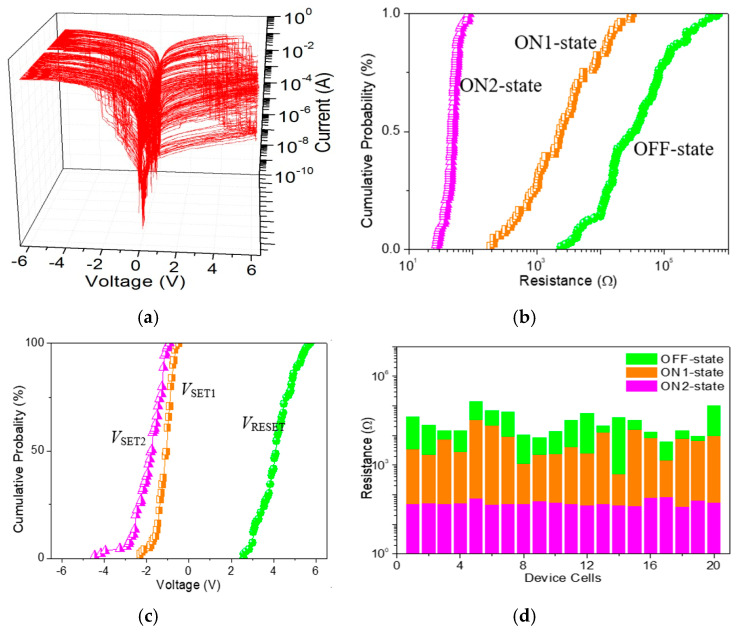
(**a**) 100 tristable sweeping cycles for ITO/GO:0.5 wt%GQDs/Ni; (**b**) Cumulative probability of the resistance in OFF, ON1 and ON2 states (*R*_OFF_, *R*_ON1_ and *R*_ON2_), and (**c**) histograms of the SET1, SET2 and RESET voltage (*V*_SET1_, *V*_SET2_ and *V*_RESET_) with respect to cycle-to-cycle operation in ITO/GO:0.5 wt%GQDs/Ni; (**d**) Device uniformity for 20 ITO/GO:0.5 wt%GQDs/Ni cells concerning *R*_OFF_, *R*_ON1_ and *R*_ON2_.

**Figure 9:**
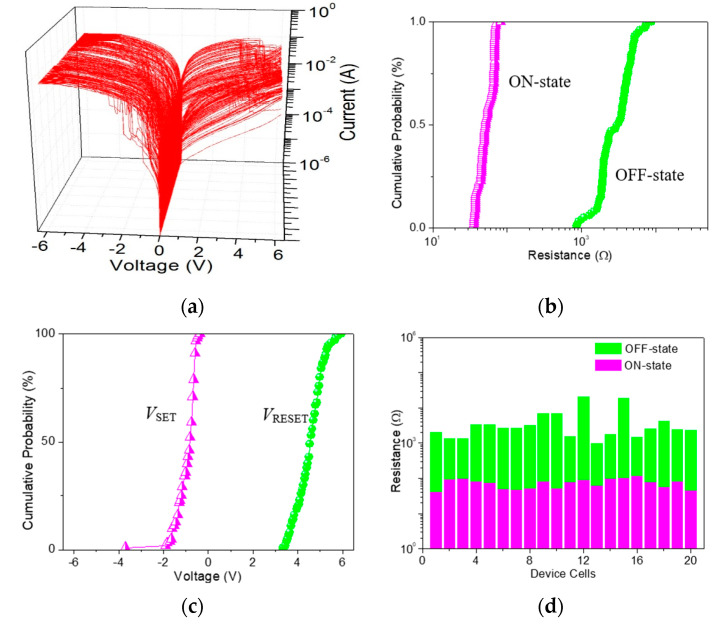
(**a**) 100 bistable switching cycles of ITO/GO/Ni; (**b**) Cumulative probability for the resistance of the device in OFF-state and ON-state (*R*_OFF_ and *R*_ON_), and (**c**) the SET and RESET voltage (*V*_SET_ and *V*_RESET_) with respect to cycle-to-cycle operation in ITO/GO/Ni; (**d**) Device-to-device uniformity for 20 device cells concerning *R*_OFF_ and *R*_ON_.

As for ITO/GO:0.5 wt%GQDs/Ni (Figure 8a), the cumulative plots of the resistance in OFF-state, ON1-state, and ON2-state (*R*_OFF_, *R*_ON1_, and *R*_ON2_), as well as histograms of *V*_SET1_, *V*_SET2_ and *V*_RESET_, demonstrate the data distributions during the cycle-to-cycle (C/C) operation (Figure 8b,c). The mean values (standard deviation) *μ*(*σ*) of *V*_SET1_, *V*_SET2_ and *V*_RESET_ were −1.12(0.37) V, −1.88(0.69) V and 4.14(0.77) V, respectively, while that of *R*_OFF_, *R*_ON1_, and *R*_ON2_ were 84.6(13.3) kΩ, 5.4(7.1) kΩ, and 48.9(11.9) Ω, respectively. For device analyses of the device-to-device (D/D) operation (Figure 8d), *μ*(*σ*) for *R*_OFF_, *R*_ON1_, and *R*_ON2_ was 36.0(34.6) kΩ, 7.5(7.8) kΩ, and 52.5(12.6) Ω, respectively. The binary memory performance of GO (Figure 9a) was meticulously studied. The cumulative plots of the resistance in OFF and ON states (*R*_OFF_ and *R*_ON_), as well as histograms of *V*_SET_ and *V*_RESET_, demonstrated the data distributions of ITO/GO/Ni during the C/C operation (Figure 9b,c). The *μ* and *σ* of *V*_SET_ were −0.95 V and 0.45 V when that of *V*_RESET_ was 4.56 V and 0.57 V. The small values of *σ* could be related to less spread in the distribution of *V*_SET_ and *V*_RESET_. Device uniformity for *R*_OFF_ and *R*_ON_ during the D/D performance (Figure 9d) indicated the value *μ*(*σ*) of *R*_OFF_ and *R*_ON_ was 3.2(1.6) kΩ and 53.8(12.1) Ω, 4.5(5.4) kΩ and 72.6(21.9) Ω for C/C and D/D operations, respectively. By doping GQDs into GO, the memory performance tunable from binary to ternary was observed to assess the possible application of the GO-based nanocomposite in all-inorganic, multi-bit resistive random-access memory (RRAM).

**Figure 10:**
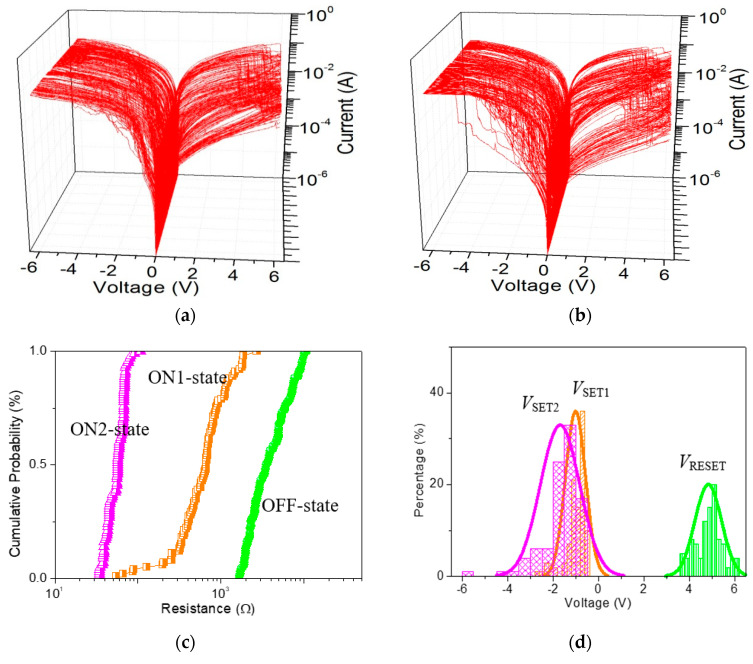

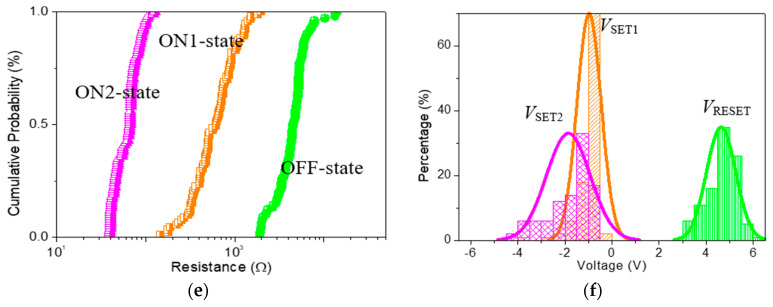
Cycle-to-cycle operation for (**a**,**b**) ITO/GO:2.5 wt%GQDs/Ni and ITO/GO:5.0 wt%GQDs/Ni; (**c**,**d**) Resistance distribution (*R*_OFF_, *R*_ON1_ and *R*_ON2_) in probability, and (**e**,**f**) *V*_SET_, *V*_SET2_ and *V*_RESET_ distributions in histogram for the cycle-to-cycle process.

To better understand the impact of GQDs on the tristable memristic switching behaviors, ITO/GO:GQDs/Ni devices with various GQDs concentrations (0.5 wt%, 2.5 wt% and 5.0 wt%) were fabricated and compared (Figure 10). Furthermore, ternary memory characteristics with the proportion *R*_OFF_/*R*_ON1_/*R*_ON2_ approaching 10^2^:10:1 were achieved by recording 100 consecutive sweeping cycles for Ni/GO:2.5 wt%GQDs/ITO and Ni/GO: 5.0 wt%GQDs/ITO. The main role of these GQDs in tristable memristic switching has to be investigated in depth. In contrast with ITO/GO: 0.5 wt%GQDs/Ni, the tristable memristic switching transition among HRS, IRS and LRS could be obviously observed under *I*_CC_ of 0.1 A when the memory cells were detected under 100 switching cycles. It could be observed with negative voltage sweeping, indicating the potential of multi-bit data storage. However, there is a significant overlap between *V*_SET1_ and *V*_SET2_, which needs to be settled in the future. The coefficient of variation (σ/μ) of *R*_OFF_, *R*_ON1_ and *V*_RESET_ reduced with incremental content of GQDs for the ITO/GO:GQDs/Ni memory cell, estimated in Table 2 and Table 3. Ternary memory behaviors with different content of GQDs (0.5 wt%, 2.5 wt% and 5.0 wt%) in the device Ni/GO:GQDs/ITO recorded that *I*-*V* curves with successive cycles were greatly stabilized by inserting GQDs into the GO layer. To summarize, all the above-mentioned results experimentally demonstrated that improved IRS and HRS robustness can be obtained by embedding GQDs in GO-based RRAM. Thus, such devices exhibit the feasibility of multi-bit RRAM.

### 3.5. Conduction Mechanism of ITO/GO-Based Film/Ni

**Figure 11:**
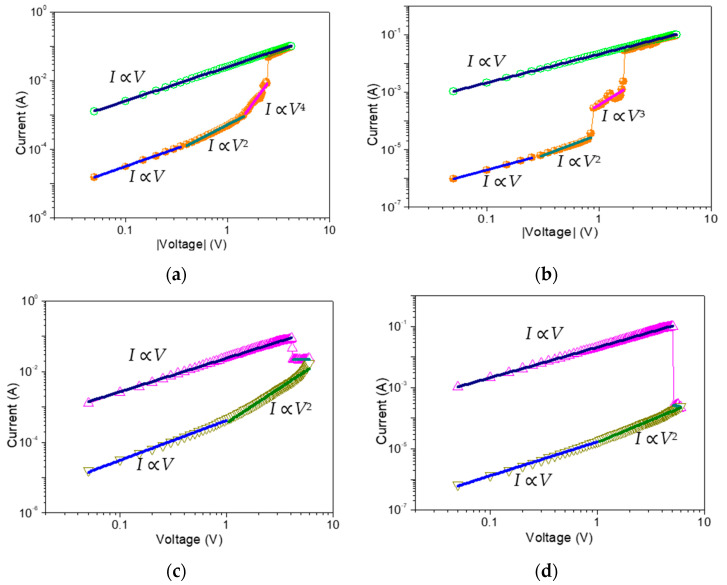
Double-logarithmic plots for (**a**,**b**) set and (**c**,**d**) reset process with the space-charge-limited model fitting for ITO /GO/ Ni and ITO /GO:0.5 wt%GQDs/ Ni, respectively.

Figure 11 illustrates a log-log plot of *I*-*V* curves on the basis of ITO/GO-based film/Ni with and without embedded GQDs. The LRS of both devices showed similar Ohmic behavior, with log*I*-log*V* slope of ~1, while HRS showed a dependence on the GQDs concentration (Table 2). For the device ITO/GO/Ni, the HRS conduction can be divided into three regions: Ohmic conduction in the low voltage region and almost linear conduction in the high voltage region with a slope of ~2 and ~4, which can be approximately linked to Ohm’s law and trap filled-limited (TFL) behavior (*I* ∝ *V*^n^) in a space charge-limited conduction model (SCLC). Herein, the injected charge carriers were believed to be captured in GO up to saturation. As the positive voltage increased, the high voltage in GO layer brought about electron detrapping. The small slope (~2) of TFL behavior in ITO/GO:0.5 wt%GQDs/Ni may be related to the shallow trapping level in the GO film. Interestingly, the slope of TFL behavior increased and saturated (a slope of ~3 in IRS of the tristable memristic switching) for the devices with embedded GQDs. It was consistent with trap-filling, which means trapping centers with deeper levels were induced by GQDs. In the low bias voltage region, the current followed Ohmic conduction (*I* ≈ *V*) because of the thermal excitation of the filled traps. With the bias growing, the traps were gradually filled. *I*-*V* curves approximately obeyed a square law (*I* ≈ *V*^2^), called by trap-filled limited current mode (TFLC). When the voltage further increased to the first threshold voltage, all traps in GO were filled up, and the conductivity of the film rapidly jumped to a higher level (“1” state). Approximately, the current was designated as space charge limited current (SCLC) region. When the voltage overcame the second threshold voltage, all traps in GO:GQDs films associated with GQDs held a stable line and the conductivity of the film rapidly jumped to a higher level (“2” state, *I* ≈ *V*^3^). The aforementioned conclusions were further corroborated upon application of both Poole–Frenkel and Schottky emission mechanisms to fit the conduction performance. The plots of log(*I*/*V*)-*V*^1/2^ and ln*I*-*V*^1/2^ before transition to Ohmic conduction were not linear and demonstrated a parabolic behavior. In summary, the impact of GQDs embedded in GO-based RRAM devices is an increase in HRS, IRS and *V*_RESET_. Since GQDs have similar chemical structures and charge trapping effects, embedding them in GO can strongly impact the electrical properties of pristine GO films and deepen the charge trapping level, which gives rise to multi-bit memristic switching behaviors.

## 4. Conclusions

In summary, memristic switching behaviors of all-inorganic multi-bit memory devices by means of the simple solution-processable method were tuned from binary to ternary through doping GQDs as charge-trapping centres into GO. An approach to improve the tristable memristic switching behaviors, such as the parameters *R*_IRS_ and *R*_OFF_ was demonstrated by the C/C operation. The coefficient of variation (*σ*/*μ*) of *R*_OFF_, *R*_ON1_ and *V*_RESET_ separately reduced to 45.09%, 55.3% and 13.3% with incremental content of GQDs for the ITO/GO:GQDs/Ni memory cell while still maintaining a resistance ratio (*R*_OFF_/*R*_ON1_/*R*_ON2_) of over 10^2^:10:1. These results provide an approach to significantly decrease memristor switching variability from cycle to cycle.

## Figures and Tables

**Table 1:** Data for FTIR spectroscopy of GO and GO:GQDs nanocomposites.

	υ_C-O-C_ (cm^−1^)	υ_C-OH_ (cm^−1^)	υ_C-C_ (cm^−1^)	υ_C=O_ (cm^−1^)	υ_OH_ (cm^−1^)
GO	1046	1338	1622	1720	3185
GO:0.5 wt%GQDs	1042	1359	1622	1714	3208
GO:2.5 wt%GQDs	1052	1377	1572	1711	3225
GO:5.0 wt%GQDs	1069	1383	1569	1696	3240

**Table 2:** Data analyses for GO:GQDs nanocomposites concerning the mean value (*μ*) and standard deviation (*σ*) of the resistance in OFF, ON1, and ON2 states (*R*_OFF_, *R*_ON1_, and *R*_ON2_).

	*R*_OFF_ (Ω)			*R*_ON1_ (Ω)			*R*_ON2_ (Ω)		
	*μ*	*σ*	*μ*/*σ*	*μ*	*σ*	*μ*/*σ*	*μ*	*σ*	*μ*/*σ*
GO:0.5 wt%GQDs	8.5 × 10^4^	1.3 × 10^5^	157.7%	5.4 × 10^3^	7.1 × 10^3^	131.3%	48.9	11.9	24.4%
GO:2.5 wt%GQDs	4.5 × 10^3^	2.6 × 10^3^	56.4%	760.3	516.7	68.0%	56.9	14.0	24.7%
GO:5.0 wt%GQDs	4.8 × 10^3^	2.2 × 10^3^	45.9%	687.9	380.3	55.3%	64.2	19.8	30.9%

**Table 3:** Values *μ* and *σ* of the SET1, SET2 and RESET voltages (*V*_SET1_, *V*_SET2_, and *V*_RESET_) based on ternary memory performance.

	*V*_SET1_ (V)			*V*_SET2_ (V)			*V*_RESET_ (V)		
	*μ*	*σ*	*μ*/*σ*	*μ*	*σ*	*μ*/*σ*	*μ*	*σ*	*μ*/*σ*
GO:0.5 wt%GQDs	−1.12	0.37	33.4%	−1.88	0.69	36.8%	4.14	0.77	18.5%
GO:2.5 wt%GQDs	−1.00	0.44	44.2%	−1.69	0.87	51.4%	4.84	0.59	12.9%
GO:5.0 wt%GQDs	−0.96	0.52	53.5%	−1.85	0.93	50.5%	4.64	0.62	13.3%
